# Cwp84, a *Clostridium difficile* cysteine protease, exhibits conformational flexibility in the absence of its propeptide

**DOI:** 10.1107/S2053230X15001065

**Published:** 2015-02-19

**Authors:** William J. Bradshaw, April K. Roberts, Clifford C. Shone, K. Ravi Acharya

**Affiliations:** a Department of Biology and Biochemistry, University of Bath, Claverton Down, Bath BA2 7AY, England; b Public Health England, Porton Down, Salisbury SP4 0JG, England

**Keywords:** *Clostridium difficile*, surface layer, Cwp84, host–pathogen interactions

## Abstract

In recent decades, the global healthcare problems caused by *Clostridium difficile* have increased at an alarming rate. A greater understanding of this antibiotic-resistant bacterium, particularly with respect to how it interacts with the host, is required for the development of novel strategies for fighting *C. difficile* infections. The surface layer (S-layer) of *C. difficile* is likely to be of significant importance to host–pathogen interactions. The mature S-layer is formed by a proteinaceous array consisting of multiple copies of a high-molecular-weight and a low-molecular-weight S-layer protein. These components result from the cleavage of SlpA by Cwp84, a cysteine protease. The structure of a truncated Cwp84 active-site mutant has recently been reported and the key features have been identified, providing the first structural insights into the role of Cwp84 in the formation of the S-layer. Here, two structures of Cwp84 after propeptide cleavage are presented and the three conformational changes that are observed are discussed. These changes result in a reconfiguration of the active site and exposure of the hydrophobic pocket.

## Abstract 2

Two structures of Cwp84, a cysteine protease from the S-layer of *C. difficile*, are presented after propeptide cleavage. They reveal the movement of three loops, two in the active-site groove and one on the surface of the lectin-like domain, exposing a hydrophobic pocket.

## 1. Introduction   

The mainly nosocomially acquired bacterium *Clostridium difficile* is the primary aetiological agent of antibiotic-associated diarrhoea, pseudomembranous colitis and toxic megacolon (Kachrimanidou & Malisiovas, 2011 ▶; Rupnik *et al.*, 2009 ▶). *C. difficile* causes tens of thousands of deaths annually and is a major global economic burden (Bouza, 2012 ▶; Dubberke & Olsen, 2012 ▶). This results in a need for further understanding of this Gram-positive bacterium, particularly towards the development of novel treatment strategies.

*C. difficile* presents a paracrystalline protein array as its outermost structure, known as a surface layer (S-layer; Fagan & Fairweather, 2014 ▶; Kawata *et al.*, 1984 ▶). The S-layer is primarily comprised of two proteins: the high-molecular-weight S-layer protein (HMW SLP) and the low-molecular-weight S-layer protein (LMW SLP), which result from the cleavage of the S-layer precursor protein SlpA (Calabi *et al.*, 2001 ▶; Cerquetti *et al.*, 2000 ▶; Karjalainen *et al.*, 2001 ▶). This cleavage is performed by Cwp84, a cysteine protease (de la Riva *et al.*, 2011 ▶; Janoir *et al.*, 2004 ▶; Kirby *et al.*, 2009 ▶) that is one of up to 28 SlpA paralogues coded for by the *C. difficile* genome (Calabi *et al.*, 2001 ▶; Fagan *et al.*, 2011 ▶). All SlpA paralogues, or Cwps (cell-wall proteins), contain three Pfam 04122 cell-wall binding domains, as seen in HMW SLP, while many also contain a ‘functional’ domain. As well as cleaving SlpA, Cwp84 has also been shown to be able to degrade the extracellular matrix proteins fibronectin, vitronectin and laminin, but is unable to break down gelatine (Janoir *et al.*, 2007 ▶).

It has previously been demonstrated that knocking out or inhibiting Cwp84 activity results in severe growth defects in *C. difficile* but is not lethal (Dang *et al.*, 2010 ▶; de la Riva *et al.*, 2011 ▶; Kirby *et al.*, 2009 ▶). This effect is likely to be due to the resultant perturbation of the S-layer structure causing stress to the bacterium, which, if it could be replicated *in vivo*, may significantly reduce the effects of *C. difficile* infection. It has been shown that a Cwp84 knockout was still able to cause symptoms in hamsters (Kirby *et al.*, 2009 ▶), but it has been speculated that perturbation of the S-layer may result in the bacterium being more susceptible to antibiotics (Dang *et al.*, 2010 ▶). A detailed understanding of the structure of the S-layer at the atomic level will be vital to causing a similar perturbation *in vivo*, preventing or reducing the severity of infections.

Recently, the crystal structure of a truncated Cwp84 active-site mutant has been reported at a resolution of 1.4 Å (Bradshaw *et al.*, 2014 ▶). Key features of the structure were identified, including the propeptide, the cysteine protease domain and the previously uncharacterized ‘lectin-like’ domain. The cysteine protease domain assumes a cathepsin L-like fold with notable differences in the occluding loop region and the proregion binding loop. The lectin-like domain, which bears significant secondary-structural and tertiary-structural similarity to carbohydrate-binding proteins, consists of a twisted β-sandwich and is stabilized by a calcium ion. The C-terminal half of the propeptide assumes a typical papain protease fold, occupying the active-site groove in the opposite direction to that of the substrate, while the N-terminal half wraps around the lectin-like domain. A method of propeptide cleavage has also been discussed (Bradshaw *et al.*, 2014 ▶).

**Figure 1:**
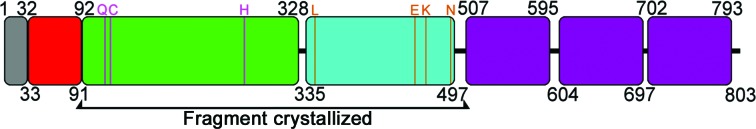
Domain representation of full-length Cwp84. The signal peptide is shown in grey, the propeptide in red, the cysteine protease domain in green, the lectin-like domain in cyan and the cell-wall-binding domains in purple. The catalytic dyad (Cys116 and His262) and oxyanion hole-forming glutamine (Gln110) are highlighted in pink, with the calcium-binding residues (Leu339, Glu448, Lys460 and Asn487) in orange. The fragment with its structure discussed here, residues 92–497, is bracketed; this complements the previously described structure consisting of residues 33–497 (Bradshaw *et al.*, 2014 ▶).

Here, two Cwp84 structures after propeptide cleavage are presented at 1.6 and 1.84 Å resolution (Fig. 1 ▶). There are at present relatively few mature cysteine protease structures in the Protein Data Bank with no ligands bound. The structures presented here therefore provide a rare opportunity to discuss the conformational changes observed in a cysteine protease upon activation. None of the conformational changes discussed appear to be shared by papain or cathepsins, the archetypes of the papain protease family.

## 2. Materials and methods   

### 2.1. Protein expression and purification   

Cwp84 was expressed and purified and the propeptide was cleaved as described previously (Bradshaw *et al.*, 2014 ▶). Briefly, a construct coding for a GST-tagged truncated Cwp84 active-site mutant, Cwp84_33–497_C116A, was expressed in LB and purified on a GST column. The tag and propeptide were removed by incubation with trypsin at a molar ratio of approximately 10:1 at 210 K for 45 min. The resulting protein, Cwp84_92–497_C116A, was further purified on a size-exclusion column, eluting in 10 m*M* Tris–HCl pH 8.0. Successful cleavage of the propeptide was confirmed by mass spectrometry and N-terminal sequencing.

### 2.2. X-ray crystallographic studies   

Crystallization conditions were screened using a Phoenix crystallization robot (Art Robbins Instruments). Crystals were observed in PACT condition D7 (0.2 *M* NaCl, 0.1 *M* Tris pH 8.0, 20% PEG 6000) and MIDAS condition F7 [20%(*v*/*v*) dimethyl sulfoxide, 20%(*v*/*v*) Jeffamine M-2070] (Molecular Dimensions). The former condition was optimized by addition of 10% Silver Bullets condition E9 [0.2%(*w*/*v*) 1,4-diaminobutane, 0.2%(*w*/*v*) cystamine dihydrochloride, 0.2%(*w*/*v*) diloxanide furoate, 0.2%(*w*/*v*) sarcosine, 0.2%(*w*/*v*) spermine, 20 m*M* sodium HEPES pH 6.8; Hampton Research]. Both crystals were cryoprotected by addition of 1 µl 50% reservoir solution and 25% glycerol.

X-ray diffraction data were collected on beamlines I04 (at 0.9795 Å wavelength) for the crystal obtained from PACT condition D7 and I04-1 (0.9200 Å wavelength) for the crystal obtained from MIDAS condition F7 at Diamond Light Source, Didcot, England. 360° of data were collected for both crystals with 0.1° oscillations. The data were autoprocessed with *XDS* (Kabsch, 2010 ▶) and *xia*2 (Winter *et al.*, 2013 ▶) and rescaled with *AIMLESS* (Evans & Murshudov, 2013 ▶), at which point 143.5° of poorer images were excluded from the MIDAS F7 data set and 60° of images were excluded from the PACT D7 data set. The structures were solved by molecular replacement with *Phaser* (McCoy *et al.*, 2007 ▶) using the previously determined Cwp84 structure (PDB entry 4ci7) with the propeptide removed as a starting model. This was followed by refinement with *Coot* (Emsley & Cowtan, 2004 ▶) and *REFMAC* (Murshudov *et al.*, 2011 ▶) and validation with *MolProbity* (Chen *et al.*, 2010 ▶).

## 3. Results   

**Figure 2:**
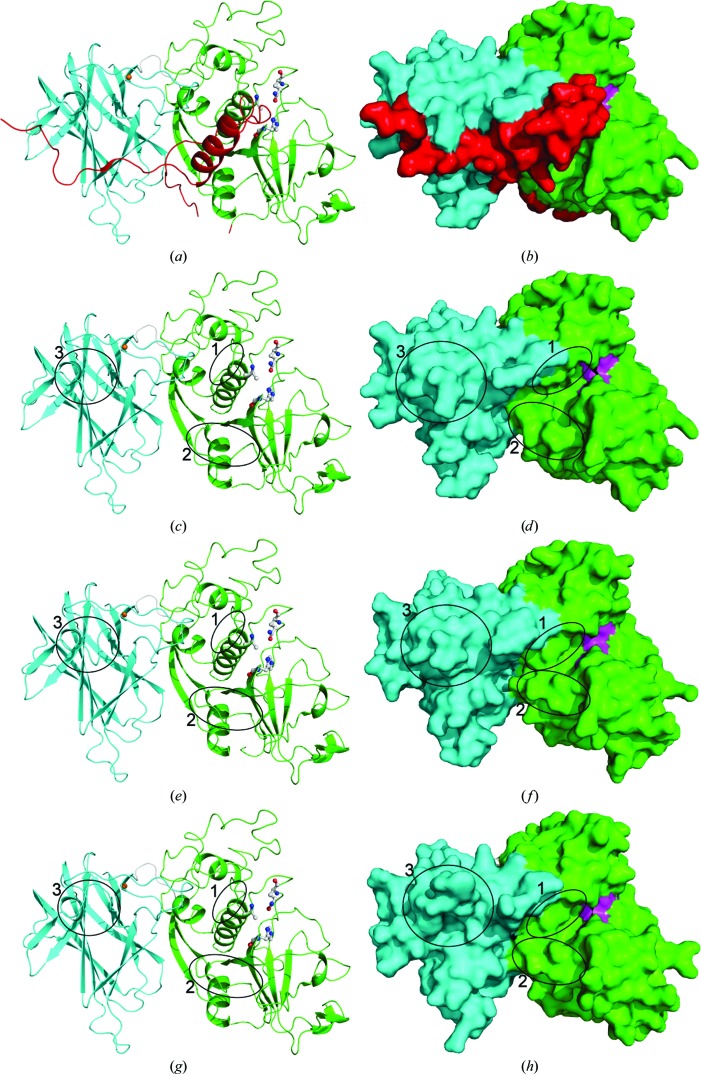
Ribbon and surface comparisons between the overall structures before and after propeptide cleavage. The domains are coloured in the same way as in Fig. 1 ▶. Gln110, C116A and His262 are shown as sticks in the ribbon diagrams and in pink in the surface representations. The calcium ion bound to the lectin-like domain is shown in orange. The three loops that undergo conformational changes are circled. 1, Met160–Ser164; 2, Leu315–Asp320; 3, Thr479–Pro485. (*a*, *b*) The previously reported structure. The propeptide interacts with both the lectin-like domain, occluding the hydrophobic pocket, and the cysteine protease domain, inserting into the active-site groove. (*c*, *d*) The same structure, but with the propeptide graphically removed, showing the shape of the active-site groove when the propeptide is bound. (*e*, *f*) Structure 1. Without the propeptide, the structure is largely unchanged except for two loops forming part of the central active-site groove; the significantly smaller S_2_ pocket is particularly notable. (*g*, *h*) The two loops in structure 2 assume the same conformation as in structure 1, while a third loop on the surface of the lectin-like domain has a vastly different conformation, exposing the hydrophobic pocket.

The structure of a truncated Cwp84 active-site mutant has been determined without the propeptide in two different sets of conditions (Fig. 2 ▶). Both sets of conditions resulted in crystallization in the triclinic space group *P*1 with two molecules in the asymmetric unit, but with different unit-cell parameters and significantly different packing of the two molecules. The structures were solved at 1.84 and 1.6 Å resolution. The lower resolution structure (from the crytal obtained using MIDAS condition F7) is referred to as ‘structure 1’, while the higher resolution structure (from the crytal obtained using PACT condition D7) is referred to as ‘structure 2’. Structure 1 contained two calcium ions, two Jeffamine molecules and 449 water molecules, while structure 2 contained four calcium ions, eight PEG molecules, four glycerol molecules and 791 water molecules. Crystallographic statistics are summarized in Table 1 ▶.

**Figure 3:**
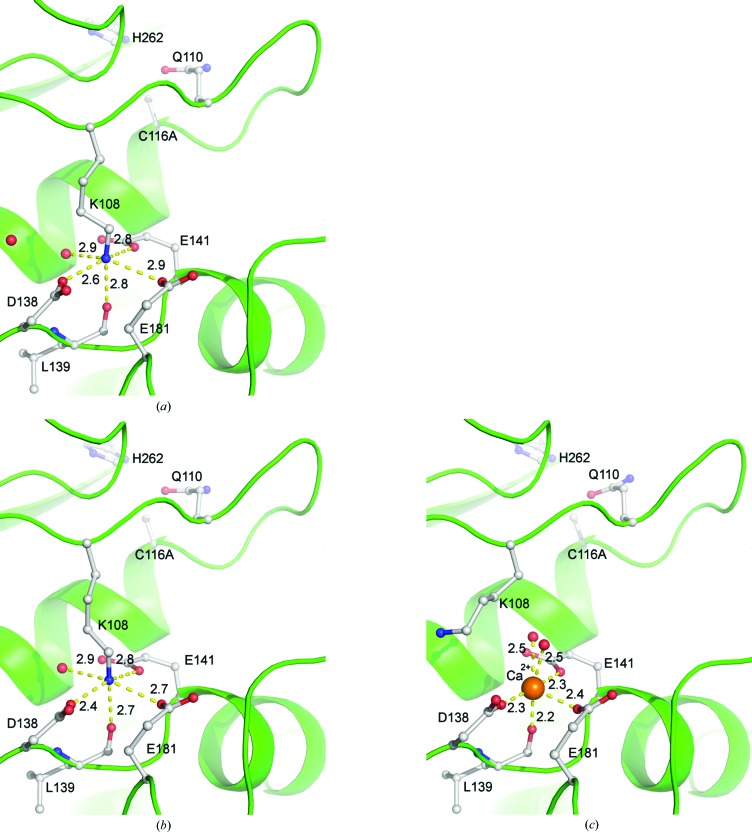
Calcium displacement of Lys108. Distances are given in Å. (*a*) The previously determined structure with the propeptide. Several negatively charged moieties bind to Lys108. (*b*) Structure 1 exhibits the same conformation as that with the propeptide. (*c*) In structure 2, Lys108 is displaced by a calcium ion. Coordination of the calcium ion is slightly tighter, but this does not result in any significant changes to the fold; notably, the nearby catalytic residues are unaffected.

As in the previous structure with the propeptide, both structures contained a calcium ion coordinated by Leu339, Glu448, Lys460 and Asn487 (Bradshaw *et al.*, 2014 ▶). The two extra calcium ions in structure 2, which are coordinated by Asp138, Leu139, Glu141 and Glu181, displace Lys108 (Fig. 3 ▶). The four coordinating residues are moderately conserved in other papain proteases (Bradshaw *et al.*, 2014 ▶; Coulombe *et al.*, 1996 ▶; Kamphuis *et al.*, 1984 ▶). Their positioning around a positively charged moiety is likely to play an important role in stabilizing the fold of the cysteine protease domain. However, the identity of this moiety does not appear to be crucial: the displacement of Lys108 by a calcium ion simply results in a different side-chain conformation. It does not appear to have a significant structural effect. All calcium-ion assignments were performed based on coordination (Zheng *et al.*, 2008 ▶) and the ability of the ion to fit the density; they were then confirmed with the *CheckMyMetal* server (Zheng *et al.*, 2014 ▶).

**Figure 4:**
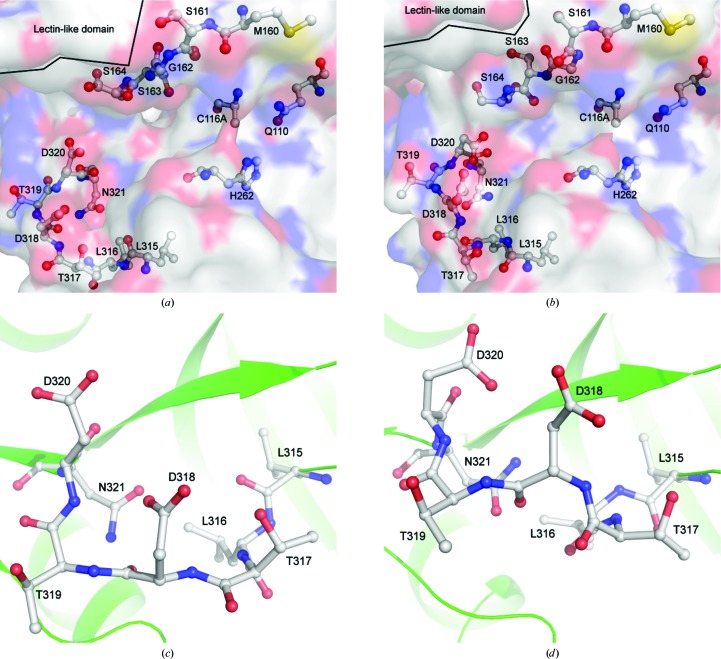
Conformational changes in the active-site groove upon propeptide cleavage. (*a*) The active-site conformation with the propeptide bound. The catalytic dyad (C116A and His262) and the oxyanion hole-forming glutamine (Gln110) are shown along with the loops formed by Met160–Ser164 and Leu315–Asp320 at the top and the bottom left, respectively. Ser161 and Ser164 exhibit multiple conformations. (*b*) The active site after propeptide cleavage. Significant rotations can be observed for Gly162 and Ser163, resulting in a change in the shape of the S_1_ pocket. This is owing to the disruption of a hydrogen-bond network with the propeptide and lectin-like domain, the latter of which is highlighted and can be seen to move away from the active-site groove. The S_2_ loop, and particularly Asp318, can be seen to move closer to the catalytic residues upon propeptide cleavage, occluding much of the previously identified P_2_ pocket. This does, however, result in a negatively charged surface formed by Asp318 and Asp320, which may be better suited to binding the P_2_ lysine of SlpA. (*c*) Close-up of the S_2_ loop before propeptide cleavage. (*d*) Close-up of the S_2_ loop after propeptide cleavage. Significant movement can be seen, particularly for Asp318.

**Figure 5:**
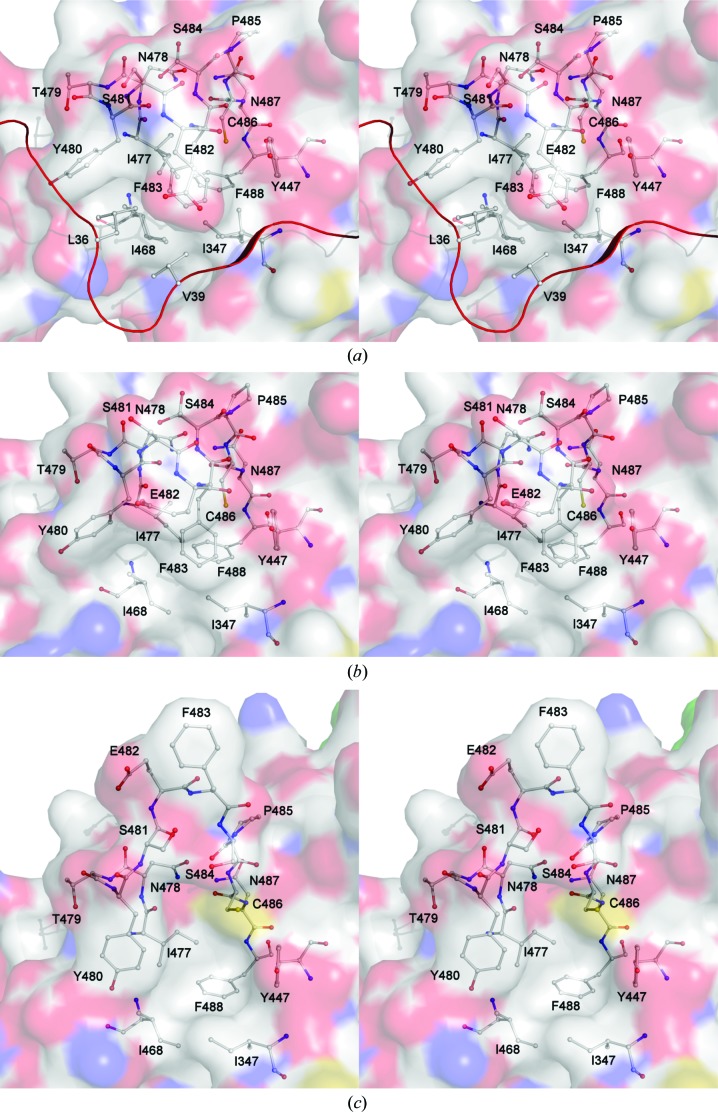
Cross-eyed stereoview of the conformational change of the hydrophobic pocket on the surface of the lectin-like domain upon propeptide cleavage. Residues forming the hydrophobic pocket and the loop formed by Thr479–Cys486 are shown. (*a*) The hydrophobic pocket with the propeptide bound; a portion of the propeptide is shown as a ribbon, with Leu36 and Val39 shown as sticks. Ile347, Ile468, Ile477 and Ser484 exhibit multiple conformations. (*b*) The hydrophobic pocket in structure 1. The conformation is similar to that with the propeptide, but the pocket is slightly more open. Ser484 exhibits multiple conformations. (*c*) The hydrophobic pocket in structure 2. The stabilizing effect of the propeptide is lost, allowing increased flexibility. This results in the pocket having greater accessibility, including the exposure of previously occluded residues.

The two structures without the propeptide are largely similar when compared to that with the propeptide (Fig. 2 ▶). The six chains (each structure contains two chains) superpose on each other with root-mean-square values of between 0.12 and 0.70 Å. There are, however, three loops that undergo notable conformational changes: Met160–Ser164 and Leu315–Asp320, which both form part of the active-site groove (Fig. 4 ▶), and Thr479–Pro485, which is located on the surface of the lectin-like domain (Fig. 5 ▶).

In the previously determined structure with the propeptide, the loop formed by Met160–Ser164, which forms the S_1_ pocket, is involved in a hydrogen-bond network with part of the propeptide (Table 2 ▶), which is supported by a high number of van der Waals interactions (Table 3 ▶; Bradshaw *et al.*, 2014 ▶). This array of noncovalent inter­actions is lost upon propeptide cleavage, allowing the backbone of Met160–Ser164 to rotate, including a rotation of approximately 160° of the peptide bond between Ser161 and Gly162. This rotation is accompanied by a movement of the loop towards the active-site residues, with a 3 Å movement of the C^α^ atom of Gly162 (Fig. 4 ▶).

Secondly, in the presence of the pro­peptide, a loop formed by Leu315–Asp320 closely interacts with the propeptide in a similar way (Tables 2 ▶ and 3 ▶; Bradshaw *et al.*, 2014 ▶). When the propeptide is cleaved, this stabilization is lost and the loop moves away from its previous position closer to the active-site groove, including a 4 Å movement of Asp318 (Fig. 4 ▶).

The cleavage of the propeptide and the conformational changes observed result in chain *A* of structure 1 exhibiting an active-site groove of 1253 Å^3^ and chain *B* of structure 2 possessing an active-site groove of 993 Å^3^, as measured by *Swiss-PdbViewer* (Guex & Peitsch, 1997 ▶). The differences in the volumes are due to slight changes in side-chain conformations. The groove narrows considerably between some of the active-site pockets, resulting in the active site being recognized as multiple separate grooves in chain *B* of structure 1 and chain *A* of structure 2.

Thirdly, a loop formed by Thr479–Pro485 is involved in the formation of the hydrophobic pocket on the surface of the lectin-like domain. With the propeptide bound, Leu36 and Val39 insert into the hydrophobic pocket, stabilizing it (Bradshaw *et al.*, 2014 ▶); this interaction is also aided by a large number of noncovalent interactions (Tables 2 ▶ and 3 ▶). After propeptide cleavage, the pocket is left somewhat more solvent-accessible, allowing the loop to become more flexible; this results in two different conformations in the structures presented here. In structure 1, apart from a slight movement away from the position of the propeptide, the conformation is largely unchanged from that seen with the propeptide, while in structure 2 the loop assumes a markedly different conformation, with the C^α^ atom of Glu482 over 9.5 Å from its previous position (Fig. 5 ▶).

## 4. Discussion   

It is known that in the C1A cysteine protease family the propeptides sit in the active-site groove in the opposite direction to the substrate (Schaschke *et al.*, 1998 ▶), allowing them to act as inhibitors (Fox *et al.*, 1992 ▶; Wiederanders, 2003 ▶). Cleavage of the propeptide reveals the active-site groove, allowing the substrate to bind. The two structures presented here complement the previously reported structure of Cwp84 with the propeptide (Bradshaw *et al.*, 2014 ▶). Together, these structures allow the identification and characterization of the conformational changes that occur upon activation of Cwp84.

Conformational changes have been described for three loops: Met160–Ser164, Leu315–Asp320 and Thr479–Pro485. If the conformational changes observed were in reality samplings of conformational space, there would be an increase in the *B* factor of the loops and different conformations may be seen in each chain. It can be concluded from this that the first two conformational changes, which show minor variations in *B* factors and no significant conformational difference between any of the four chains presented here, are conformational changes that occur upon propeptide cleavage. Conversely, owing to more significant increases in *B* factors and different conformations being observed for the third loop in the two structures, this is more likely to represent a loss of conformational stability caused by the loss of the stabilizing effect of the propeptide on the hydrophobic pocket.

The first two conformational changes described, those of Met160–Ser164 and Leu315–Asp320, result in a reconfiguration of the active site. The former is likely to be involved in formation of the S_1_ pocket, while the latter is likely to form the S_2_ pocket. There are no symmetry-related molecules in close proximity to either of these loops in either chain of any of the three structures, so they are unlikely to be crystallographic artefacts.

The rotation of Met160–Ser164 appears to result in a fairly minor change to the surface of the S_1_ pocket (Fig. 4 ▶). The shape is very similar; however, the previously occluded C^α^ atom of Gly162 produces a small hydrophobic patch and the side chain of Ser163 points into the active-site groove rather than away from it. These changes, particularly the movement of Ser163, may result in the stabilization of the conserved P_1_ serine of SlpA (Qazi *et al.*, 2009 ▶). Comparing this with the archetypal C1A cysteine proteases, the loop does not appear to exhibit any conformational change that is likely to be significant upon propeptide cleavage in papain (Kamphuis *et al.*, 1984 ▶; Roy *et al.*, 2012 ▶), cathepsin L (Adams-Cioaba *et al.*, 2011 ▶; Coulombe *et al.*, 1996 ▶) or cathepsin B (Musil *et al.*, 1991 ▶; Podobnik *et al.*, 1997 ▶; Turk *et al.*, 1996 ▶).

The conformation of the S_2_ pocket observed in the present structures is likely to be unfavourable when the propeptide is bound, as Asp318 would lie 2.5–3.0 Å from Pro68, resulting in hydrophobic/hydrophilic repulsion. The result of this is that Leu315–Asp320 are further away from the active-site residues when the propeptide is bound. This conformation is then stabilized by a range of noncovalent interactions, producing the conformation seen in the previously reported structure with the propeptide intact (Bradshaw *et al.*, 2014 ▶).

The stabilization of this conformation will be significantly weakened upon cleavage of the propeptide, allowing the loop to move closer to where Pro68 would have been, forming the mature S_2_ pocket seen in the present structures. The movement of this loop results in the occlusion of the majority of the previously described S_2_ pocket (Fig. 4 ▶; Bradshaw *et al.*, 2014 ▶), leaving Asp318 and Asp320 forming a negatively charged patch to which the P_2_ lysine of SlpA can bind.

This loop does not show any movement upon propeptide cleavage in papain (Kamphuis *et al.*, 1984 ▶; Roy *et al.*, 2012 ▶) or cathepsin B (Musil *et al.*, 1991 ▶; Podobnik *et al.*, 1997 ▶; Turk *et al.*, 1996 ▶), while in cathepsin L there is some movement, particularly for Ser213, but the S_2_ selectivity residue, Ala214, does not move (Adams-Cioaba *et al.*, 2011 ▶; Coulombe *et al.*, 1996 ▶).

Thr479–Pro485, the residues involved in the third conformational change, form part of the previously described hydrophobic pocket on the surface of the lectin-like domain, into which Leu36 and Val39 from the propeptide insert (Bradshaw *et al.*, 2014 ▶). Upon removal of the propeptide, all residues forming the pocket become somewhat more accessible, potentially reducing the ability of the hydrophobic effect to drive the conformation of the loop.

In structure 1, this loop assumes a similar closed conformation to when the propeptide is present, but is likely to be more flexible. This is shown by the slightly weaker electron density and resultant higher *B* factors. The loop adopts a vastly different, much more open conformation in structure 2 (Fig. 5 ▶), exposing the hydrophobic pocket. There are also a small number of interactions involving two symmetry-related molecules, which are likely to have an influence on the side-chain orientations of Tyr480 and Phe483 and potentially stabilize the open conformation. However, it is likely that this conformation is only permitted by the loss of the closed-conformation stabilization of the propeptide.

## 5. Conclusions   

Two structures of Cwp84 without its propeptide have been determined, complementing the previously determined structure with the propeptide intact. Together, these structures allow the identification and discussion of structural changes that occur upon propeptide cleavage. Propeptide cleavage causes two loops which form the S_1_ and S_2_ pockets of the active-site groove to undergo conformational changes, resulting in a reconfiguration of the pockets. The new conformations seen in the present structures are likely to facilitate binding of the P_1_ and P_2_ residues of SlpA: serine and lysine, respectively. A third loop in structure 2, found on the surface of the lectin-like domain, also exhibits a conformational change. This exposes the hydrophobic pocket that was previously occluded by the propeptide. This conformation appears to be somewhat stabilized by crystal contacts. Nonetheless, the same loop in structure 1 does assume a slightly more open conformation than when the propeptide is bound. The structural data presented here provide a detailed molecular basis for the role of the propeptide of Cwp84.

## Supplementary Material

PDB reference: Cwp84 after propeptide cleavage, structure 1 at 1.84 Å, 4d59

PDB reference: structure 2 at 1.60 Å, 4d5a

## Figures and Tables

**Table 1:** X-ray crystallographic statistics Values in parentheses are for the outer shell.

	Structure 1	Structure 2
Space group	*P*1	*P*1
Unit-cell parameters		
*a* ()	42.2	48.1
*b* ()	58.4	70.2
*c* ()	93.1	78.9
()	89.3	65.2
()	78.0	89.9
()	71.6	80.2
Resolution range ()	55.41.84	47.31.60
*R*_merge_ (%)	7.1 (58.9)	9.2 (46.7)
*I*/(*I*)	13.6 (2.7)	6.8 (2.1)
Completeness (%)	83.5 (83.0)	90.8 (49.9)
No. of reflections	155876 (9319)	313196 (7586)
Unique reflections	59711 (3659)	110175 (2994)
Multiplicity	2.6 (2.5)	2.8 (2.5)
Wilson *B* factor (^2^)	19.4	7.8
*R*_cryst_/*R*_free_ (%)	22.7/29.1	18.1/21.0
Average *B* factor (^2^)		
Overall	32.0	16.1
Protein	31.9	14.7
Ligand	31.6	30.3
Solvent	32.0	26.5
R.m.s. deviations		
Bond lengths ()	0.009	0.009
Bond angles ()	1.323	1.209
Ramachandran plot statistics (%)		
Preferred	96.5	95.4
Allowed	3.5	4.6
Disallowed	0	0
PDB code	4d59	4d5a

**Table 2:** Hydrogen bonds between the propeptide and the mature protein Bond-distance ranges quoted are the distances seen in the two chains of the previously published structure, except where specified (Bradshaw *et al.*, 2014 ▶). The majority of the hydrogen bonds seen are near to one of the three conformational changes described. Roughly, the first six are near the hydrophobic pocket, the next six are near the S_1_ pocket and the remaining 15 are near the S_2_ pocket. Charge-based interactions of less than 3.2 in length are listed.

Propeptide	Atom	Mature protein	Atom	Distance ()
Lys34	NZ	Thr479	OG	2.722.77
Gly38	O	Ser349	OG	2.772.95
Glu40	O	Met348	N	2.832.86
Glu40	N	Met348	O	2.832.92
Thr41	OG	Tyr447	OH	2.552.56
Ala42	N	Lys346	O	3.083.15
Tyr63	O	Tyr455	OH	2.253.00
Asn64	O	Asn114	ND	2.763.12
Gly65	O	Ser163	N	3.143.24
Val66	N	Leu260	O	3.053.08
Ile67	N	Ser163	O	2.822.84
Ile67	O	Ser164	OH	3.00†
Met73	O	Thr139	OG	2.772.81
Glu74	N	Asp375	OD2	2.812.83
Glu74	O	Ser409	OG	2.672.72
Glu74	OE1	Glu441	OE1	3.05†
Glu74	OE2	Glu441	OE1	2.95†
Thr76	OG	Ser409	N	2.842.85
Thr76	OG	Arg215	O	2.782.82
Thr76	N	Arg215	O	2.792.86
Thr76	O	Asn217	N	2.92‡
Thr77	OG	Asn217	O	2.742.83
Leu78	N	Thr222	OG	2.983.03
Leu78	O	Asn225	ND	2.983.06
Arg79	NH1	Asp219	OD2	2.853.04
Arg79	NH2	Asp219	OD2	3.153.34
Arg79	NH2	Thr222	OG	3.063.13

† Glu74 and Ser164 assume slightly different conformations in chain *B*, so the hydrogen bonds are not present. Either Glu74 or Glu441 must be protonated for a hydrogen bond to be present.

‡ The bond length is 2.92 in both chains.

**Table 3:** Van der Waals interactions between the propeptide and the mature protein As in Table 2 ▶, the majority of interactions seen are near to one of the three conformational changes described. The table shows three ‘peaks’ approximately centred on Val39, Ile67 and Thr76; these peaks correspond to the hydrophobic pocket, the S_1_ pocket and the S_2_ pocket, respectively, although part of the second peak can be attributed to residues involved in the formation of the S_2_ pocket. Residues were considered to be in van der Waals contact if the distance between any of their constituent atoms was less than 4.2.

Propeptide	Mature protein
Lys34	Thr479, Tyr480
Thr35	Tyr480
Leu36	Ile468, Gln470, Ile477, Tyr480, Phe483
Asp37	Gln470
Gly38	Ser349
Val39	Ile347, Met348, Ser349, Ile468, Glu482, Phe483
Glu40	Ile347, Met348, Glu482, Phe483
Thr41	Ser345, Lys346, Met348, Tyr447, Phe483
Ala42	Ser345, Lys346, Met348,
Tyr44	Ile451, Asp452
Tyr48	Asp452, Tyr454
Tyr51	Tyr455
Leu52	Gly453
Ala61	Pro259
Lys62	Pro259
Tyr63	Gly162, Tyr455
Asn64	Asn114, Met160, Ser161, Gly162, Tyr455
Gly65	Gly162, Ser163, Leu360
Val66	Ser163, Leu260, Asn261
Ile67	Ser163, Ser164, Tyr454, Tyr455, Leu456
Phe69	Val166, Arg215, Asp320, Tyr322, Leu344
Pro70	Leu344, Ile451, Tyr455
His71	Leu344, Ser345, Ile451
Glu72	Lys346
Met73	Arg215, Thr319, Gly343, Ser345, Lys346, Asp375
Glu74	Arg215, Asp375, Ser409, Glu441
Gly75	Arg215, Asn217, Thr319, Ser409
Thr76	Val214, Arg215, Leu216, Asn217, Thr222, Tyr408, Ser409
Thr77	Asn217, Thr222
Leu78	Leu216, Glu221, Thr222, Asn225, Ala226, Tyr230
Arg79	Asp219, Glu221, Thr222, Asn225
